# Alterations in pelvic kinematics with speed, incline, and fatigue in female runners

**DOI:** 10.3389/fspor.2025.1721641

**Published:** 2026-01-15

**Authors:** Jaka Kovše, Irinej Papuga, Miha Drobnič, Ahsen Buyukaslan, Nina Verdel, Vojko Strojnik, Matej Supej

**Affiliations:** Faculty of Sport, University of Ljubljana, Ljubljana, Slovenia

**Keywords:** asymmetry, biomechanics, mixed effect model, pelvic kinematics, rearfoot strike, running related injury

## Abstract

**Introduction:**

In running, female runners show higher overuse-injury rates, partly due to sex-specific anatomy and biomechanics. Pelvic motion is central to lower-limb kinematics, however, female-specific responses are underexamined. This study tested how running speed, incline, and fatigue influence pelvic rotation, tilt, and obliquity in recreational female runners.

**Methods:**

Twenty-two females completed treadmill trials at 10, 12, and 14 km/h on level ground and at 10 km/h with 5% and 10% inclines, before and after a 30-minute run at 80% heart-rate reserve to induce moderate fatigue. A 3D motion-capture system recorded pelvic rotation, tilt, and obliquity at heel-strike, toe-off, peak values, and ranges of motion. Linear mixed-effects models assessed main and interaction effects; asymmetry was quantified via symmetry index between left and right gait cycles.

**Results:**

Higher speeds increased peak pelvic rotation, tilt, and obliquity, and enlarged rotation and obliquity range of motion. A 10% incline raised peak pelvic obliquity and rotation and increased range of motion for rotation, tilt, and obliquity; a 5% incline had no measurable effect. Fatigue increased peak pelvic rotation and range of motion for rotation and tilt. A fatigue × 10% incline interaction showed that incline-related increases in tilt range of motion observed when fresh were reduced under fatigue. Pelvic tilt asymmetry rose with speed.

**Discussion:**

Speed, incline, and fatigue each modulate pelvic kinematics in recreational female runners, with effect sizes often exceeding reports from mixed-sex samples. Notably, greater frontal-plane motion at higher speeds and increased transverse-plane motion with incline and fatigue may heighten loads on the iliotibial band, hamstrings, or lumbar spine.

## 1 Introduction

Running is very popular in Europe, with an estimated participation of approximately 50 million individuals (1). Its popularity is likely attributable to the established physical and physiological health benefits associated with running (2–4). Nonetheless, running is correlated with high annual injury rates, ranging from 20% to 70% (5), frequently associated with high weekly training volumes and insufficient running experience (6, 7).

Female runners exhibit a disproportionate prevalence of specific overuse injuries, including patellofemoral pain, iliotibial band syndrome, and tibial stress fractures, with relative risk estimates reaching up to twice those observed in male runners (8–12). These disparities may be partially explained by anatomical sex-based variations, such as reduced hip musculature strength (13–15), greater hip-width-to-thigh-length ratio (16), greater hip abduction (16), and hip internal rotation (16, 17), as well as greater trunk flexion (13) and trunk rotation compared to male runners (13, 17). These anatomical characteristics often contribute to increased hip internal rotation and adduction (16, 17), alongside increase in knee valgus during gait cycles (18).

Although the majority of running-related injuries (RRIs) are localised to the knee and lower foot complex (7, 19), approximately one-third involve the hip, pelvis, or lumbar spine (20). The pelvis modulates the kinematic behaviour of the knee and ankle joints by serving a critical stabilizing function within running biomechanics, thereby facilitating efficient energy transfer between the lower extremities and the upper body (21). Moreover, evidence suggests that pelvic kinematic abnormalities can alter spinal biomechanics (22) and influence distal lower limb structures (15), which may result in increased mechanical stress and elevated injury susceptibility. Variations in pelvic orientation, such as tilt, rotation, or obliquity, have been associated with specific injury types, including hamstring strains, lumbar pain, tibial stress fractures, iliotibial band syndrome, calf strains, patellofemoral pain syndrome, and sacroiliac joint injuries (23–28).

From a neuromechanical perspective, increased running speed may induce alteration in pelvic motion patterns attributable to heightened joint loading (29, 30). Running uphill is expected to shift mechanical work from the knee to the hip by increasing hip extensor moments, which may result in changes to pelvic control strategies (31–33). Furthermore, fatigue has been shown to reduce trunk-pelvis-hip coordination and increase kinematic variability during running, suggesting that fatigued runners may exhibit greater pelvic ranges of motion and altered timing of pelvic motion (34–36).

Previous investigations have explored the effects of walking and running speed (29, 37), slope incline (37), and fatigue (34, 35) on pelvic kinematics, however, the majority of these studies either aggregated data across sexes or primarily focused on male participants. When female subjects were included, analyses seldom examined sex-specific differences in kinematic parameters such as magnitude and variability or their implications for injury risk modulation. For instance, Chumanov et al. (37) reported higher peak pelvic obliquity angles in females compared to males at various speeds and inclines but did not extend these observations to potential sex-dependent injury mechanisms.

This study aims to fill this gap by examining the effects of running speed, incline, and fatigue on pelvic kinematics, including potential asymmetries between left and right strides, exclusively within a population of recreational female runners. Furthermore, it delineates female-specific biomechanical responses that may vary in magnitude or clinical significance relative to mixed-sex cohorts. Finally, the study evaluates potential implications for injury prevention strategies tailored to female runners, considering that pelvic control mechanisms and susceptibility to injury may diverge from those observed in male populations.

## 2 Materials and Methods

### 2.1 Participants

A total of twenty-five female recreational runners were enrolled in this study. The cohort had a mean age of 26.9 years (±11.9 years), a mean body mass of 59.1 kg (±5.2 kg), a mean height of 167.4 cm (±3.9 cm), and a mean weekly running distance of 18.5 km (±13.0 km) per week. The age distribution was positively skewed, with a greater prevalence of younger individuals*.* Participant recruitment was conducted through the Faculty of Sports and affiliated local sports clubs, with eligibility criteria determined via a standardised questionnaire.

Inclusion criteria required participants to be habitual heel-strikers, engage in a minimum of five hours of physical activity per week, have no history of neurological or chronic non-communicable diseases, and be free from injuries for at least six months preceding participation. All participants provided written informed consent prior to enrolment. The study was approved by the Committee of Ethical Issues in Sports at the University of Ljubljana, Slovenia (Approval Number: 1/2023).

### 2.2 Study design

Participants attended initial laboratory session during which their heel-strike running pattern was evaluated while running on a treadmill at speeds of 10 and 14 km/h, under both level and inclined conditions. Individuals meeting the eligibility criteria subsequently proceeded to execute the standardised testing protocol.

**Figure 1:**
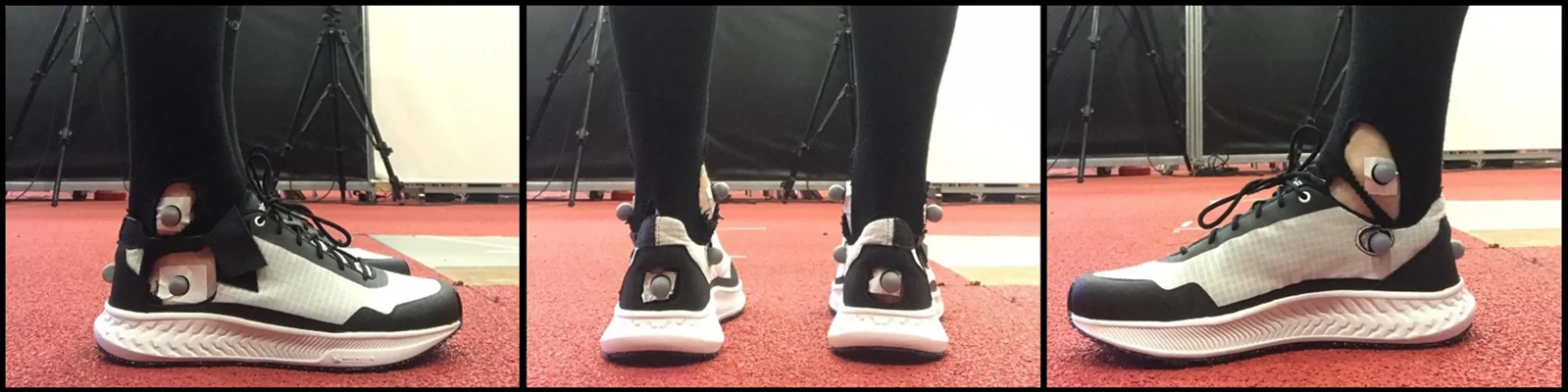
Modified Alpina Madeira running shoes featuring openings on the sides and heel to accommodate placement of motion capture markers directly on the skin.

Resting heart rate (HRmin) was measured via a Polar H10 chest strap heart rate monitor (Polar Electro Oy, Kempele, Finland) prior to the initiation of the running protocol. Maximum heart rate (HRmax) was calculated using the formula: HRmax = 220—age (38). Participants were provided with a specially designed shoe model, Alpina Madeira (Alpina, Žiri, Slovenia), tailored to their appropriate sizes. The footwear was equipped with strategically positioned openings (Figure 1) allowing for the direct attachment of motion markers to the skin, thereby ensuring a more precise representation of foot kinematic data than marker placement on the shoe surface. Prior to testing, participants had the opportunity to familiarise themselves with both the footwear and the treadmill during a warm-up phase, consisting of a light 5-min jog at self-selected speed.

The running protocol consisted of two sets of five randomized one-minute treadmill runs, separated by a fatiguing protocol. The five treadmill runs included running at speeds of 10, 12, and 14 km/h, as well as at 10 km/h with inclines of 5% and 10%. A standardised 1-min rest interval was implemented between each running condition in both sets. Given the submaximal intensity of the one-minute exercise bouts and the uniform duration of work-rest intervals, a 1-min rest interval was considered sufficient to reduce immediate carry-over fatigue during the initial set. However, following the fatiguing protocol, the same 1-min rest period was not long enough to allow full recovery between efforts, as evidenced by sustained elevated fatigue levels reported by participants.

According to ACSM guidelines exercise intensity is classified as high at 60%–85% of heart rate reserve (HRR) (39). Based on these guidelines and previous report of high ratings of perceived exertion during 30-min runs at similar intensities (40), a 30-min treadmill run at 80% HRR was employed as the fatiguing protocol. Target HR was calculated using the Karvonen formula: target HR = [(HRmax—HRmin) × 0.8] + HRmin. HR was continuously monitored throughout the exercise, and participants adjusted their running pace as necessary to maintain the target HR. This methodology, grounded in Karvonen's method (41), provides a more individualised assessment of exercise intensity compared to a percentage of HRmax alone. Participants were unable to maintain a constant running velocity at 80% HRR, indicating this level of exertion was sufficiently vigorous to induce both cardiovascular and neuromuscular fatigue.

Immediately after the 30-min run, participants reported subjective fatigue using Visual Analogue Scale (VAS) score, where 0 indicated “no fatigue’ and 10 “extreme fatigue”. The mean VAS score following the fatiguing protocol was 6.27 ± 0.88, reflecting moderate fatigue. Participants then completed a second set of five randomized one-minute treadmill runs identical to initial set. VAS scores were reassessed immediately afterward, yielding a mean of 6.26 ± 1.3. The lack of significant change (*p* = 0.49) suggests that participants remained in a moderately fatigued state, and that the one-minute recovery intervals were insufficient for full recovery post-fatigue.

VAS was chosen over the Rating of Perceived Exertion, as it is designed to assess perceived fatigue following exercise and offers a continuous, highly sensitive measurement. Previous studies have shown that VAS scores correlate with physiological indicators of fatigue such as lactate accumulation and heart rate changes (42, 43), and that VAS ratings are intuitive even for individuals with limited experience using exertion scales (44). In contrast, the reliability of RPE appears to depend more heavily on prior familiarity with the scale, which may be limited in recreational runners (44).

### 2.3 Equipment

The protocol for the ongoing study was conducted on a TRX Marathon 3.0 treadmill (Toorx, Pazzolo Formigaro, Italy), featuring a running belt measuring 530 × 1,520 mm, with an adjustable speed range of 0.8–22.0 km/h and an incline capability of up to 13%. Heel-strike events were recorded using a high-frequency camera (DS-CAM−1,100 m) operating at 300 frames per second, in conjunction with an 8-channel, 24-bit, 200 kHz Dewe−43A DAQ system, all controlled via DewesoftX software (all DEWESoft, Trbovlje, Slovenia).

**Figure 2:**
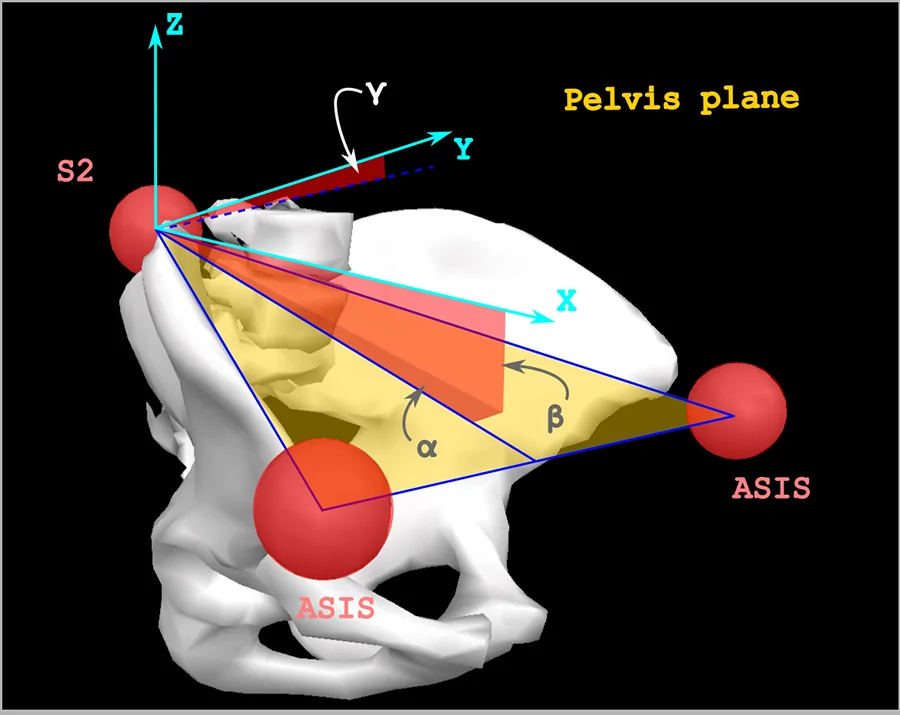
A three-dimensional model of the pelvic region is presented, with the global coordinate system. The spheres located on the anterior superior iliac spine (ASIS) landmarks and the spinous process of the second sacral vertebra (S2) landmark represent anatomical markers, which define the pelvic plane. The rotational parameters of the pelvis are defined by three angular measurements: pelvic rotation (*α*), pelvic tilt (*β*), and pelvic obliquity (*γ*). These angles are determined based on the orientation of the pelvic plane in relation to the global coordinate system.

Kinematic data were collected using the Qualisys Oqus motion capture system, consisting of 12 infrared cameras (Qualisys AB, Gothenburg, Sweden) sampling at a rate of 180 Hz. Reflective markers were strategically placed on participants in accordance with the Qualisys Sports Marker Set, with partial adherence to the IORfoot Marker Set for precise step detection. Additional markers were affixed to the treadmill frame for calibration purposes. Pelvic kinematics were derived from markers positioned at key anatomical landmarks, specifically the anterior superior iliac spine (ASIS) and the spinous process of the second sacral vertebra (S2), as depicted in Figure 2. Landmark identification and marker placement were performed following protocols outlined in the Color Atlas of Skeletal Landmark Definitions *(45)*.

### 2.4 Data analysis

Kinematic data for the entire lower kinetic chain were processed using Qualisys Track Manager software and subsequently exported to Visual3D software (C-Motion, Maryland, USA) for the computation of rigid body orientations. No trajectory filtering procedures were implemented within Qualisys, as filtering has the potential to distort high-frequency components of the signal and consequently diminish the temporal accuracy of heel-down event detection. MATLAB R2024b (MathWorks, Massachusetts, USA) was employed to implement algorithms for detecting heel-strike and toe-off events (73).

The primary kinematic parameters analysed, as depicted in Figure 2, included pelvic rotation (*α_0_*), tilt (*β_0_*), and obliquity (*γ_0_*) at heel-strike and at toe-off (*α_to_*, *β_to_*, *γ_to_*), along with their respective maximum values (*α_max_*, *β_max_*, *γ_max_*) and the corresponding range of motion (ROM) metrics (*α_rom_*, *β_rom_*, *γ_rom_*), defined as the difference between the minimum and maximum angles. Data from three participants were excluded due to incomplete or invalid recordings, resulting in a final sample size of 22 participants. For each participant, an average of 67.2 ± 16.6 heel-strike events per leg per run were successfully recorded and precisely labelled, culminating in approximately 22,500 steps analysed.

### 2.5 Statistical analysis

Separate linear mixed-effects models were employed for each kinematic metric (pelvic angle) to account for inter-subject variability and to address the heteroscedasticity observed in the residual plots. The analysis evaluated the effects of running speed, incline, and fatigue on these metrics, including their interaction effects. Initial data review revealed some asymmetries between left and right steps; therefore, limb side and its interactions with speed and incline were incorporated as factors in the models. Participants were modelled as random effects to appropriately handle heteroscedasticity. The final models for each kinematic metric can be expressed as: kinematicmetric∼speed*fatigue+incline*fatigue+incline *limbside+speed*limbside

Further analyses investigated potential asymmetries in pelvic angles during left and right steps, utilising the symmetry index (SI) (74) with the absolute difference in the denominator to quantify asymmetries in rotation, tilt, and obliquity at key gait events: heel-strike, toe-off, peak rotation, and range of motion. SI values approaching 0% indicate high symmetry, whereas values approaching 100% suggest complete asymmetry. All statistical computations were performed using the statsmodels and scipy libraries within Python 3.12.

## 3 Results

The influence of varying running conditions on pelvic kinematics is detailed in Tables 1, 2. Table 1 specifies the mean pelvic angle values, accompanied by standard deviations, for all participants under different speed, incline, and fatigue conditions. Table 2 presents the differences relative to the primary reference condition (running at 10 km/h on a 0% incline before the fatigue protocol) and subsequent conditions, including associated *p*-values and 95% confidence intervals (CI).

**Table 1:** Three-dimensional kinematics of the pelvis during running under different conditions of speed, incline and fatigue.

Kinematic parameter	10 km/h	12 km/h	14 km/h	10 km/h	10 km/h
	0% incline	0% incline	0% incline	5% incline	10% incline
*α*_0_ [ °]	−2.0 ± 4.1 −2.2 ± 4.3	−2.2 ± 4.3 −2.2 ± 4.7	−1.5 ± 4.3 −2.0 ± 4.3	−2.3 ± 4.1 −2.7 ± 4.4	−3.2 ± 4.4 −3.5 ± 4.2
α_to_ [ °]	2.6 ± 3.6 2.8 ± 4.0	2.9 ± 4.0 3.2 ± 4.4	2.6 ± 4.1 3.0 ± 4.2	3.0 ± 3.8 3.1 ± 4.2	3.5 ± 3.6 4.2 ± 4.1
α_max_ [ °]	6.1 ± 3.5 7.5 ± 4.0*	6.8 ± 3.6 8.6 ± 4.3*	7.6 ± 3.5 9.0 ± 3.5*	6.6 ± 3.4 7.4 ± 3.9*	6.8 ± 3.6 7.8 ± 3.7*
α_rom_ [ °]	14.2 ± 4.0 16.0 ± 4.1*	15.8 ± 4.3 18.3 ± 4.9*	17.0 ± 4.3 19.3 ± 4.6*	14.8 ± 3.3 16.0 ± 3.9*	15.2 ± 3.3 16.7 ± 3.9*
*β*_0_ [ °]	20.6 ± 4.4 20.1 ± 4.3*	21.4 ± 4.5 20.6 ± 4.5*	21.2 ± 4.6 20.7 ± 4.5	20.3 ± 4.4 19.6 ± 4.3*	20.2 ± 4.8 19.2 ± 4.2*
β_to_ [ °]	21.2 ± 4.3 20.8 ± 4.0*	22.3 ± 4.3 21.6 ± 4.4	22.1 ± 4.6 21.8 ± 4.6	21.2 ± 4.4 20.3 ± 4.4*	21.1 ± 4.9 20.1 ± 4.4*
β_max_ [ °]	22.6 ± 4.4 22.1 ± 4.3*	23.8 ± 4.4 23.1 ± 4.4*	24.0 ± 4.4 23.5 ± 4.4	22.4 ± 4.4 21.7 ± 4.5*	22.4 ± 4.8 21.5 ± 4.5*
β_rom_ [ °]	8.2 ± 1.8 8.7 ± 1.8*	8.7 ± 1.8 8.8 ± 1.6	8.6 ± 1.5 8.9 ± 1.5	8.5 ± 1.7 8.7 ± 1.6	8.6 ± 1.8 8.6 ± 1.6
*γ*_0_ [°]	−3.2 ± 2.4 −3.4 ± 2.8	−3.5 ± 2.5 −3.6 ± 2.9	−3.3 ± 2.5 −3.8 ± 2.4*	−3.4 ± 2.5 −3.8 ± 2.6	−4.3 ± 2.5 −4.5 ± 2.6
γ_to_ [°]	4.6 ± 2.5 4.7 ± 2.7	5.7 ± 2.6 6.1 ± 2.6*	6.5 ± 2.7 6.9 ± 2.3	4.8 ± 2.5 4.9 ± 2.5	5.3 ± 2.7 5.3 ± 2.6
γ_max_ [°]	7.5 ± 2.6 7.8 ± 2.9	8.3 ± 2.5 8.5 ± 3.0	8.9 ± 2.6 8.9 ± 2.4	7.6 ± 2.5 7.8 ± 2.5	8.0 ± 2.5 8.2 ± 2.6
γ_rom_ [°]	14.0 ± 4.1 14.5 ± 3.5*	15.5 ± 4.0 16.0 ± 3.6*	16.5 ± 3.7 17.0 ± 3.6*	14.3 ± 3.7 14.1 ± 2.9	15.3 ± 3.6 15.1 ± 2.9

α_0…_pelvic rotation at heel-strike, α_to…_pelvic rotation at toe off, α_max…_peak pelvic rotation, α_rom…_pelvic rotation range of motion, β_0…_pelvic tilt at heel-strike, β_to…_pelvic tilt at toe off, β_max…_peak pelvic tilt, β_rom…_pelvic tilt range of motion, γ_0…_pelvic obliquity at heel-strike, γ_to…_pelvic obliquity at toe off, γ_max…_peak pelvic obliquity, γ_rom…_pelvic obliquity range of motion.

Values are presented as mean ± standard deviation in degrees. For each pelvic variable, the first row represents the mean value before the onset of fatigue, and the second row represents the mean value after the onset of fatigue. Statistically significant differences (*p* < 0.05) between moderately fatigued and non-fatigued running conditions are marked with *.

**Table 2:** Results of linear mixed models. Each row represents a model that was used for a specific pelvic angle.

Kinematic parameter	12 km/h	14 km/h	5% incline	10% incline	Fatigued
α_0_ [ °]	0.02 °, *p* = 0.97, CI = [−1.62, 1.67]	0.63 °, *p* = 0.46, CI = [−1.03, 2.28]	−0.28 °, *p* = 0.74, CI = [−1.94, 1.37]	−1.18 °, *p* = 0.16, CI = [−2.83, 0.48]	−0.22 °, *p* = 0.75, CI = [−1.57, 1.13]
α_to_ [ °]	0.45 °, *p* = 0.57, CI = [−1.09, 1.99]	0.24 °, *p* = 0.76, CI = [−1.30, 1.78]	0.37 °, *p* = 0.64, CI = [−1.17, 1.91]	1.04 °, *p* = 0.18, CI = [−0.50, 2.58]	0.23 °, *p* = 0.72, CI = [−1.03, 1.49]
α_max_ [ °]	0.70 °*, *p* = 0.03, CI = [0.07, 1.34]	1.54 °*, *p* < 0.01, CI = [0.90, 2.17]	0.54 °, *p* = 0.10, CI = [−0.10, 1.17]	0.76 °*, *p* = 0.02, CI = [0.13, 1.40]	1.43 °*, *p* < 0.01, CI = [0.92, 1.94]
α_rom_ [ °]	1.47 °*, *p* < 0.01, CI = [0.72, 2.23]	2.80 °*, *p* < 0.01, CI = [2.04, 3.55]	0.51 °, *p* = 0.19, CI = [−0.25, 1.26]	0.98 °*, *p* = 0.01, CI = [0.23, 1.74]	1.75 °*, *p* < 0.01, CI = [1.14, 2.37]
β_0_ [ °]	0.82 °*, *p* = 0.02, CI = [0.12, 1.51]	0.48 °, *p* = 0.18,CI = [−0.22, 1.17]	−0.29 °, *p* = 0.42, CI = [−0.98, 0.41]	−0.36 °, *p* = 0.31, CI = [−1.06,0.33]	−0.55 °, *p* = 0.06, CI = [−1.11, 0.02]
β_to_ [ °]	0.99 °*, *p* < 0.01, CI = [0.32, 1.66]	0.88 °*, *p* = 0.01, CI = [0.20, 1.55]	−0.17 °, *p* = 0.62, CI = [−0.84, 0.50]	−0.27 °, *p* = 0.44, CI = [−0.93,0.41]	−0.49 °, *p* = 0.08, CI = [−1.03, 0.07]
β_max_ [ °]	1.17 °*, *p* < 0.01, CI = [0.56, 1.78]	1.40 °*, *p* < 0.01, CI = [0.79, 2.01]	−0.19 °, *p* = 0.53, CI = [−0.80, 0.42]	−0.23 °, *p* = 0.46, CI = [−0.84, 0.38]	−0.49 °, *p* = 0.05,CI = [−0.98, 0.01]
β_rom_ [ °]	0.45 °*, *p* = 0.02, CI = [0.06, 0.84]	0.36 °, *p* = 0.07, CI = [−0.03, 0.76]	0.26 °, *p* = 0.19, CI = [−0.13, 0.66]	0.40 °*, *p* = 0.04, CI = [0.01, 0.80]	0.53 °*, *p* < 0.01, CI = [0.21, 0.84]
γ_0_ [ °]	−0.29 °, *p* = 0.59, CI = [−1.33, 0.75]	−0.03 °, *p* = 0.95, CI = [−1.08, 1.01]	−0.19 °, *p* = 0.72, CI = [−1.24, 0.85]	−1.42 °*, *p* = 0.03, CI = [−2.19, −0.10]	−0.17 °, *p* = 0.70, CI = [−1.02, 0.69]
γ_to_ [ °]	1.10 °*, *p* = 0.04, CI = [0.03, 2.17]	2.03 °*, *p* < 0.01, CI = [0.96, 3.10]	0.27 °, *p* = 0.62, CI = [−0.80, 1.34]	0.90 °, *p* = 0.10, CI = [−0.17, 1.97]	0.15 °, *p* = 0.73, CI = [−0.73, 1.03]
γ_max_ [ °]	0.76 °*, *p* < 0.01, CI = [0.28, 1.24]	1.38 °*, *p* < 0.01, CI = [0.90, 1.86]	0.06 °, *p* = 0.81, CI = [−0.42, 0.51]	0.51 °*, *p* = 0.04, CI = [0.03, 0.99]	0.26 °, *p* = 0.19, CI = [−0.13, 0.65]
γ_rom_ [ °]	1.49 °*, *p* < 0.01, CI = [0.82, 2.16]	2.48 °*, *p* < 0.01, CI = [1.81, 3.15]	0.30 °, *p* = 0.38, CI = [−0.37, 0.97]	1.33 °*, *p* < 0.01, CI = [0.66, 2.00]	0.51 °, *p* = 0.06, CI = [−0.03, 1.06]

α_0__…_pelvic rotation at heel-strike, α_to…_pelvic rotation at toe off, α_max…_peak pelvic rotation, α_rom…_pelvic rotation range of motion, β_0…_pelvic tilt at heel-strike, β_to…_pelvic tilt at toe off, β_max…_peak pelvic tilt, β_rom…_pelvic tilt range of motion, γ_0…_pelvic obliquity at heel-strike, γ_to…_pelvic obliquity at toe off, γ_max…_peak pelvic obliquity, γ_rom…_pelvic obliquity range of motion.

The models were modelled after (Equation 1). Results are presented as an average difference relative to primary reference condition (running at 10 km/h on 0% incline before the fatigue protocol), *p*-value and 95% confidence interval. Statistically significant results (*p* < 0.05) are marked with *.

### 3.1 Influence of speed

Pelvic rotation at heel-strike (*α₀*) and toe-off (*α_to_*) and pelvic obliquity at heel-strike (*γ₀*) were not significantly affected by increasing running speed (Table 2). Meanwhile, pelvic tilt at heel-strike (*β₀*) and tilt range of motion (*β_rom_*) increased at 12 km/h but showed no further increases at 14 km/h. In contrast, maximum pelvic rotation (*α_max_*), peak pelvic tilt (*β_max_*), and peak pelvic obliquity (*γ_max_*) increased significantly at 12 and 14 km/h compared to primary reference condition. Similarly, the ranges of motion for pelvic rotation (*α_rom_*) and pelvic obliquity (*γ_rom_*), as well as pelvic tilt (*β_to_*) and obliquity at toe-off (*γ_to_*), showed significant increases at 12 and 14 km/h compared to 10 km/h.

### 3.2 Influence of incline

As shown in Table 2, none of the pelvic angles were affected by a 5% incline. In contrast, running at a 10% incline produced significant changes compared to level running, with a slight increase in *α_max_*, *β_rom_*, and *γ_0_*, and moderate increases in *α_rom_, γ_max_*, and *γ_rom_* while *α₀*, *β₀*, *α_to_*, *β_to_*, *γ_to_* and *β_max_* remained unaffected.

### 3.3 Influence of fatigue

According to reported VAS scores, participants achieved only moderate fatigue. Despite this, significant increases in *α_max_* and *β_rom_* were observed when running in a moderately fatigued state (Table 2). A slightly smaller but still significant increase was observed by *β_rom_* in fatigued state as well. In contrast, *α_0_*, *α_to_*, *β_to_*, *γ_to_* *γ_max_*, and *γ_rom_* were not affected by fatigue. Similarly, decreases in *β_0_*, *β_max_*, and *γ*_0_ were observed, but these changes were too small to be considered significant.

### 3.4 Interaction of factors

No significant interaction effects between speed and fatigue were observed for any of the pelvic angles considered. Similarly, no significant interaction effects between incline and fatigue were found for all parameters except one. A significant negative interaction for *β_rom_* [*−0.5 °, p* *=* *0.02, CI* *=* *(−1.0, −0.01)*] was observed at 10% incline. Comparable results were found for interaction effects between speed and limb side, where no significant effects were observed. However, a significant negative interaction between incline and limb side was detected at 10% incline for *α_0_* and *γ_0_*, which decreased by −2.7 ° [*p* *<* *0.01, CI* *=* *(−4.6, −0.7)*] and −2.3 ° [*p* *<* *0.01, CI* *=* *(−3.5, −1.2)*], respectively.

### 3.5 Asymmetries

Regarding *α_0_* and *α_to_*, pelvic rotation was notably asymmetrical at primary running condition (*SI* *=* *52.4%* and *51.3%,* respectively), which were not significantly influenced by any experimental condition. There was considerable variability between subjects. For *α_max_*, baseline asymmetry was minimal (*SI* *=* *2.75%*), and a significant improvement in symmetry was observed at a running speed of 14 km/h compared to 10 km/h [*SI* *=* *−1.64%, p* *=* *0.02, CI* *=* *(−2.98, −0.31)*]. Additionally, fatigue was associated with a significant improvement in *α_max_* symmetry [*SI* *=* *−1.74%, p* *=* *0.01, CI* *=* *(−3.08, −0.40)*]. The symmetry of *α_rom_* was initially high (*SI* *=* *0.85%*), and a significant improvement was observed under fatigue condition [*SI* *=* *−0.52%, p* *=* *0.01, CI* *=* *(−0.93, −0.12)*], whereas neither speed nor incline had significant effects.

*β_0_* and *β_to_* asymmetries were modest (*SI* *=* *2.65%,* and *1.84%,* respectively) during primary running conditions, with a notable increase in *β_0_* only observed at 12 km/h [*SI* *=* *1.02%, p* *=* *0.03, CI* *=* *(0.10, 1.95)*]. No significant effects were identified for 14 km/h, fatigue, or incline. Additionally, *β_to_* showed no significant effects under any running condition. Concerning *β_max_*, which exhibited high symmetry at primary running condition (*SI* *=* *0.31%*) a slight increase in symmetry was observed at 14 km/h [*SI* *=* *−0.15%, p* *=* *0.03, CI* *=* *(−0.28, −0.01)*], while a slight decrease was observed at 10% incline [*SI* *=* *0.17%, p* *=* *0.01, CI* *=* *(0.04, 0.31)*]. Pelvic tilt range of motion was highly symmetrical at the primary running condition (*SI* *=* *0.33%*), however, symmetry decreased significantly at 10% incline [*SI* *=* *0.40%, p* *<* *0.01, CI* *=* *(0.14, 0.65)*]. Speed and fatigue did not produce any significant effects on this parameter.

Pelvic obliquity at heel-strike and at toe-off exhibited notable asymmetry during the primary running condition (*SI* *=* *51.0%* and *35.7%,* respectively). The asymmetry in *γ_0_* significantly decreased at 10% incline [*SI* *=* *−14.1%, p* *=* *0.01, CI* *=* *(−24.07, −4.15)*] and at 14 km/h in the fatigued state [*SI* *=* *−14.7, p* *=* *0.04, CI* *=* *(−28.78, −0.62)*]. Similarly, *γ_to_* asymmetry showed significant reductions at both 12 [*SI* *=* *−9.2%, p* *<* *0.05, CI* *=* *(−18.10, −0.21)*] and 14 km/h and [*SI* *=* *−10.4%, p* *=* *0.02, CI* *=* *(−19.37, −1.48)*]. There was substantial variability observed across participants. For *γ_max_* and *γ_rom_*, pelvic obliquity was nearly symmetrical during primary running condition (*SI* *=* *0.61%*, *SI* *=* *0.30%*, respectively) and was not significantly influenced by any of the experimental conditions.

## 4 Discussion

This study examined how running speed, incline, and moderate fatigue independently relate to pelvic kinematics in recreational female runners, using step-level kinematic data (∼22,500 steps) and linear mixed-effects models. Higher speeds were associated with increases in peak pelvic rotation, tilt, and obliquity, pelvic tilt at heel-down and toe-off, as well as larger rotation and obliquity range of motion. Speed was also related to decrease in pelvic obliquity at heel-down. A1 0% incline produced small-to-moderate increases in obliquity at heel-strike, peak obliquity and the ranges of motion of rotation, tilt, and obliquity, whereas 5% incline had negligible effects. Fatigue modestly increased peak rotation and the ranges of motion of rotation and tilt. An incline-fatigue interaction indicated that the tilt range of motion increase seen at 10% incline when fresh was attenuated under fatigue. Asymmetry effects were generally small and variable, with large SI values at heel-strike and toe-off likely reflecting denominator sensitivity near zero.

Collectively, the data support a measured conclusion: in women, faster running and steeper uphill grades are linked to modest increases in frontal and transverse pelvic motion, and moderate fatigue primarily amplifies rotation-related measures.

### 4.1 Influence of speed

The hypothesis that increased running speed would increase peak pelvic rotation, tilt and obliquity, as well as ranges of motion for rotation and obliquity, while concurrently decreasing tilt range of motion, was partially supported. Consistent with prior research involving mixed-sex and male-dominant populations (29, 37), our female cohort demonstrated significant increases in peak pelvic rotation, tilt, and obliquity, as well as rotation and obliquity range of motion, with higher speeds. The extent of these changes, however, exceeded those reported in earlier work, suggesting that female runners may display a more pronounced kinematic response to speed increases. For example, Chumanov et al. (37) observed only a 0.7° increase in peak pelvic obliquity when increasing speed from 9.7 to 13 km/h, whereas our data showed a 1.4° increase from 10 to 14 km/h; nearly double the angular change, despite only a 20% difference in speed range. Additionally, our observed obliquity range of motion increased by 2.5°, compared to just 1.1° in Chumanov's findings over a similar speed range. These differences may reflect greater frontal-plane mobility in females, potentially related to sex-specific pelvic anatomy and hip abductor function (16).

Perpiñá-Martínez et al. (47) reported that female runners generally exhibit higher baseline values for pelvic rotation, tilt, and obliquity compared to male runners at similar moderate running speeds (8.75–10.11 km/h). Additionally, these angles tend to increase with running speed. In the current study, similar trends were observed; however, the baseline pelvic angle values at 10 km/h were higher than those reported by Perpiñá-Martínez et al., indicating greater frontal-plane motion and increased sagittal-plane range of motion than previously documented. These variations may be attributable to differences in running speed, data collection methods, and participants wearing unfamiliar, specially designed footwear. The higher obliquity values in this cohort, along with the more pronounced increases with speed compared to both Chumanov et al. and Perpiñá-Martínez et al., suggest that female heel-strikers may adapt differently to increased running speeds. These differences could be influenced by factors such as pelvic morphology, stride mechanics, and muscle activation patterns (46, 48, 49).

**Figure 3:**
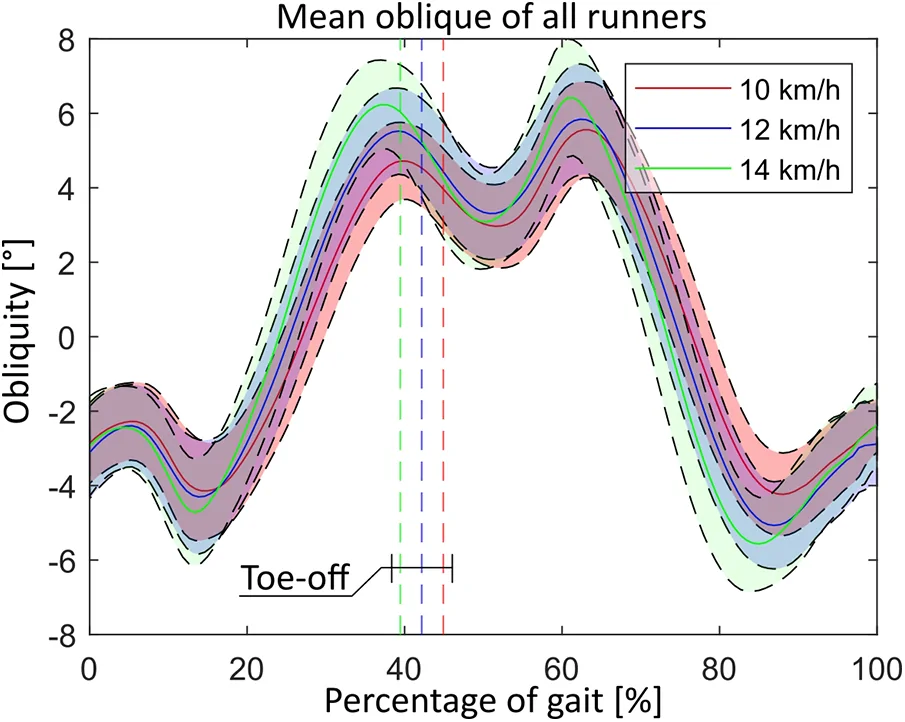
Mean (± standard deviation) pelvic obliquity trajectory across all participants. Solid lines represent the mean for each running speed, and shaded areas indicate the corresponding standard deviation. Vertical dashed lines mark toe-off at each speed. With increasing speed, stance time shortens and toe-off occurs earlier in the gait cycle, aligning more closely with the first obliquity peak.

In addition to the observed increases in peak pelvic kinematics at higher running speeds, an increase in pelvic tilt and obliquity at toe-off was also noted. Increased pelvic tilt may reflect a greater forward trunk-pelvis lean in the sagittal plane, which can facilitate the redirection of ground reaction forces more horizontally and potentially enhance running speed (29). In Figure 3, both a timing-related effect and an increase in pelvic obliquity across the stance phase are apparent. As running speed increases, ground contact time generally decreases (50), leading to an earlier occurrence of toe-off within the gait cycle. Consequently, toe-off at higher speeds corresponds to a different portion of the pelvic obliquity waveform than at lower speeds, which should be considered when interpreting discrete toe-off values across conditions.

This increased magnitude of change associated with higher running speed, as well as higher initial values of pelvic angles in females compared to males could have important implications for injury risk. Increased pelvic obliquity and tilt at faster paces have been linked to elevated loads on the iliotibial band, hamstrings, and lumbar spine (23, 51, 52). Biomechanically, greater pelvic obliquity at higher speeds has been associated with narrower step width and longer stride length, which have been shown to reduce gluteus medius preactivation and compromise frontal-plane pelvic stability (53, 54). Although this mechanism has been proposed based on mixed-sex samples, the higher injury prevalence observed in women and the greater pelvic angles identified in our study indicate that increases in pelvic obliquity related to speed may have greater significant clinical implications for female runners. From a preventative perspective, female runners should prioritize targeted strengthening exercises for the hip abductors and core musculature, as well as implement gait modifications that reduce stride length and modestly elevate cadence. These interventions are correlated with decreased contralateral pelvic drop and hip adduction during locomotion (53, 55–57).

### 4.2 Influence of incline

It was hypothesised that increasing the incline would affect pelvic kinematics, and this was partially supported by the findings. Elevating the incline from 0% to 10% resulted in significant decrease in pelvic obliquity at heel-strike, indicating a greater deviation from neutral frontal-plane, such as increased contralateral pelvic drop. Additionally, it resulted in increases in peak pelvic obliquity, as well as the range of motion for pelvic obliquity and pelvic tilt. These findings expand on previous research by Chumanov et al. (37), which observed greater pelvic obliquity in females compared to males during uphill running. The current findings demonstrate that incline running in women also elicits increases in peak pelvic rotation and the ranges of rotational and tilt motions. A direct comparison with the study by Chumanov et al. (37) highlights notable differences: their data indicated that peak pelvic obliquity increased in both males and females, with both groups exceeding the increases found here. It is worth noting that baseline peak obliquity values in their cohort were lower than those in the current participants, suggesting that participants in their study started with higher obliquity levels and may have had less potential for further increase.

The observed increases in pelvic motion in this study can be explained through biomechanical principles: as the incline increases, the ground reaction force shifts anterior to the hip's axis of rotation, resulting in a longer lever arm at the hip and shorter lever arm at the knee. This redistribution of mechanical work, places greater demands on the hip joint while reducing the load on the knee (31). In response to elevated joint loading, especially when the knee extensor muscles are unable to adequately regulate motion, force generation is increasingly delegated to the stronger gluteal musculature (58). A marginal anterior tilt of the trunk and pelvis positions the hip extensor muscles within a more advantageous length-tension relationship for force generation (59). Furthermore, increased pelvic motion in the sagittal and frontal planes can diminish the necessity for extensive knee flexion during uphill locomotion, thereby biomechanically shifting the primary load-bearing role proximally from the knee to the hip (33). In the context of this study, these alterations in load distribution are likely to have directly influenced pelvic kinematics, as evidenced by the significant increases in pelvic obliquity, tilt, and rotation observed during incline running.

Unexpectedly, notable increases in both peak pelvic rotation and pelvic rotation range of motion were observed at 10% incline, suggesting that incline running affects a wider range of pelvic angles than previously anticipated. The observed increase in hip extension during uphill running (60) may potentially contribute to increased pelvic rotation as a compensatory mechanism to maintain stride length, however, existing research does not yet provide conclusive evidence of a consistent relationship.

### 4.3 Influence of fatigue

The hypothesis regarding the impact of fatigue was only partially supported. Fatigue was associated with an increase in peak pelvic rotation, pelvic rotation range of motion, and tilt range of motion. However, unlike Borba et al. (34), no significant reductions in peak pelvic tilt or tilt at heel-strike were observed. Instead, an increase in tilt range of motion was identified, which was not reported in their study using a mixed, but male-dominant cohort. Additionally, the observed increases in maximum pelvic rotation and rotation range of motion were more pronounced than those reported by Borba et al.

No significant increase in the obliquity range of motion was observed, which contrasts with the findings of Maas et al. (35), who reported greater obliquity range of motion in a mixed cohort of runners following a 3,200 m maximal effort run. This discrepancy may be attributed not only to higher fatigue levels in their protocol but also to the analysis being conducted on a mixed cohort rather than sex-specific groups, which may have obscured sex-related differences in pelvic kinematic responses. Overall, these findings suggest that certain pelvic kinematic changes, particularly tilt at heel-strike, peak tilt, and obliquity range of motion, may require higher levels of fatigue to manifest. In contrast, rotation parameters and tilt range of motion appear to have greater sensitivity in female runners compared to males and can be affected under moderate fatigue conditions.

### 4.4 Influence of fatigue and incline interaction

Since the runners did not run on different inclines at higher speeds of 12 and 14 km/h, a fully balanced experimental design for the incline and speed factors could not be established. Therefore, only interactions between speed and fatigue, incline and fatigue, speed and limb side, and incline and limb side could be examined to assess potential asymmetries. A significant interaction between fatigue and incline was observed for pelvic tilt range of motion. The data indicated that pelvic tilt range of motion was affected by incline running in a manner that depended on fatigue status. Specifically, in a non-fatigued state, a 10% incline was associated with an increase in pelvic tilt range of motion; however, under fatigue, this increase was not observed and was slightly reversed. Supporting this finding, Tazji et al. (36) demonstrated that fatigue reduces coordination and increases variability in trunk-pelvis-hip coupling during treadmill running, implying that neuromuscular control of pelvic motion becomes compromised under fatigue. This interaction suggests that while incline running may promote greater pelvic tilt range of motion when fresh, fatigue limits the ability to adapt kinematics to terrain changes, potentially impacting efficiency and increasing mechanical load on adjacent joints or the lumbar spine.

### 4.5 Asymmetries in pelvic kinematics

Significant asymmetries in pelvic rotation and obliquity at heel-strike as well as at toe-off were observed at initial running condition (running at 10 km/h on flat). No significant effects of different running conditions were observed for pelvic rotation at heel-strike. However, asymmetry in pelvic obliquity at heel-strike was significantly reduced at 10% incline and when running at 14 km/h in a fatigued state, while at toe-off asymmetry reduced significantly with increasing speed. The observed large baseline asymmetries may be attributable to methodological factors, such as potential errors in automatic heel-strike and toe-off detection, variability in marker placement, or the sensitivity of the SI to small denominators when left and right angles are near zero (61). Employing alternative measures, such as the Symmetry Angle, may help address these limitations. Other pelvic angles exhibited a high degree of symmetry, with condition effects resulting in only minor variations. Although some of these effects were statistically significant, their magnitude was limited.

To our knowledge, limited research has examined asymmetries in pelvic motion during running. Lin et al. (62) reported significant pelvic rotation asymmetry at toe-off, which was not reported in this study. Most existing work has instead focused on asymmetries in cadence, contact time, and ground reaction forces. The present findings may therefore encourage further investigation into pelvic kinematic symmetry and its potential implications for running-related injuries.

### 4.6 Implications

These findings suggest that speed, incline and fatigue each contribute to increased pelvic motion in the frontal and transverse planes in females, sometimes exceeding levels observed in mixed-sex cohorts. Given the higher prevalence of patellofemoral pain, iliotibial band syndrome and tibial stress fracture among female runners (9, 10, 12), these adaptations may carry greater clinical implications. Increased pelvic obliquity indicated at higher speeds and steeper inclines has been associated with sacroiliac joint injury, iliotibial band syndrome, patellofemoral pain and with greater lateral knee loading (23, 54, 63–65). Greater pelvic tilt has been linked to increased tissue elongation across the hamstring muscles and higher risk of low back pain, hamstring strain and calf muscle injury (24, 26, 27, 51, 52). Reduced peak pelvic rotation has been associated with a greater likelihood of tibial stress fractures (25, 32), whereas runners with low back pain have been reported to exhibit increased pelvic rotation range of motion compared to asymptomatic controls (28), suggesting that altered transverse-plane pelvic control in either direction may be important. Present results revealed that higher speeds were associated with greater pelvic obliquity and tilt, steeper inclines contributed to greater pelvic rotation and obliquity, and fatigue amplified the range of motion of pelvic rotation and tilt, potentially reflecting diminished core and hip stability (15). Speed, incline and fatigue related changes in pelvic kinematics observed in the present study may reflect neuromechanical configurations consistent with proposed mechanisms for patellofemoral pain, iliotibial band syndrome, tibial stress fracture and other common overuse injuries in female runners.

Based on the current findings, several practical considerations can be proposed. First, high-speed running should be performed sparingly and kept relatively short in duration, as it produces the largest pelvic excursions and imposes substantial mechanical demands on the hip and trunk musculature. Sustaining such intensities for extended periods, without adequate recovery, may amplify cumulative neuromuscular fatigue and elevate injury risk**.** Additionally, adjustments to running biomechanics can aid in minimizing excessive pelvic displacement: decreasing stride length and elevating cadence diminish impact transient forces and pelvic oscillations (53, 55), while adopting a forefoot or midfoot strike pattern may further attenuate ground-reaction forces (66). Reduced impact magnitudes decrease the necessity for compensatory pelvic movements, consequently, surface and footwear choices are integral factors influencing load absorption and enhancing pelvic stability and control (67, 68). Although uphill running can decrease impact loading, downhill running significantly increases impact forces, potentially elevating the risk of overuse injuries (69). Therefore, we recommend selecting steeper inclines for uphill and gentler gradients for downhill running. Lastly, incorporating strength training exercises targeting the core and lower extremities is recommended, as it enhances resistance to fatigue and promotes more stable running biomechanics (70).

The presented results underscore the significance of monitoring pelvic kinematics in runners to improve our understanding of injury mechanisms and to devise targeted strategies for injury prevention and rehabilitation. Building on this, machine learning-based feature extraction has recently been used to characterize gait and detect subtle condition-related changes in both sports and clinical contexts. Such approaches may reveal complex spatiotemporal signatures that are difficult to capture with discrete variables alone and have been proposed as valuable tools for gait pattern recognition and injury risk assessment (71). While this study used standard 3D motion capture, the pelvic kinematic adaptations observed across speed, incline, and fatigue could serve as candidate features for future data-driven models of gait pattern recognition and injury risk assessment. Future research should prioritise interventions that aim to optimise running mechanics, such as strength training and gait modifications, to reduce the risk of running-related injuries.

### 4.7 Limitations

While this study offers valuable insights several limitations should be acknowledged. The sample consisted exclusively of recreational female runners, with ages ranging from 19 to 58 years (mean age: 27.4 years ± 11.5 years), with a distribution skewed toward younger participants. This demographic likely represents the most active segment of the female running population. Consequently, the findings may not be applicable to runners from different age groups or those with alternative training backgrounds, such as competitive athletes or elderly. Furthermore, although more than 90% recreational runners tend to be heel-strikers (72), this study exclusively examined individuals with a heel-to-toe foot strike pattern. Including midfoot and forefoot strikers in future research would enhance our understanding of how foot strike patterns influence pelvic kinematics. The study design did not systematically combine all levels of speed, incline, and fatigue conditions, which limits the ability to analyse potential interaction effects among these variables. Finally, the fatigue protocol utilised a 30-min run, which may not fully replicate the fatigue levels experienced during longer or more intense running sessions, and fatigue was inferred from moderate VAS fatigue score rather than direct physiological measures such as blood lactate. To further validate and expand these findings, future studies should consider more diverse samples, incorporate all foot strike patterns, test all factor combinations, employ varied fatigue protocols and include physiological markers for stronger objective confirmation of fatigue level.

## Data Availability

The datasets presented in this study can be found in Repository of University of Ljubljana (RUL) at the following link https://repozitorij.uni-lj.si/IzpisGradiva.php?id=167830&lang=slv#datoteke.

## References

[1] Breedveld K Scheerder J Borgers J. Running across Europe. In: Running Across Europe. (2015) 1:1–27. 10.1057/9781137446374.0017

[2] Lee IM Shiroma EJ Lobelo F Puska P Blair SN Katzmarzyk PT. Effect of physical inactivity on major non-communicable diseases worldwide: an analysis of burden of disease and life expectancy. Lancet. (2012) 380(9838):219–29. 10.1016/S0140-6736(12)61031-9 22818936 PMC3645500

[3] Oswald F Campbell J Williamson C Richards J Kelly P. A scoping review of the relationship between running and mental health. Int J Environ Res Public Health. (2020) 17(21):1–39. 10.3390/ijerph17218059 PMC7663387 33139666

[4] Sallis R Franklin B Joy L Ross R Sabgir D Stone J. Strategies for promoting physical activity in clinical practice. Prog Cardiovasc Dis. (2015) 57(4):375–86. 10.1016/j.pcad.2014.10.003 25459975

[5] Ferber R Hreljac A Kendall KD. Suspected mechanisms in the cause of overuse running injuries: a clinical review. Sports Health. (2009) 1(3):242–6. 10.1177/1941738109334272 23015879 PMC3445255

[6] Buist I Bredeweg SW Bessem B Van Mechelen W Lemmink KAPM Diercks RL. Incidence and risk factors of running-related injuries during preparation for a 4-mile recreational running event. Br J Sports Med. (2010) 44(8):598–604. 10.1136/bjsm.2007.044677 18487252

[7] Van Gent RN Siem D Van Middelkoop M Van Os AG Bierma-Zeinstra SMA Koes BW. Incidence and determinants of lower extremity running injuries in long distance runners: a systematic review. Br J Sports Med. (2007) 41(8):469–80). 10.1136/bjsm.2006.033548 17473005 PMC2465455

[8] Boling M Padua D Marshall S Guskiewicz K Pyne S Beutler A. Gender differences in the incidence and prevalence of patellofemoral pain syndrome. Scand J Med Sci Sports. (2010) 20(5):725–30. 10.1111/j.1600-0838.2009.00996.x 19765240 PMC2895959

[9] Dempster J Dutheil F Ugbolue UC. The prevalence of lower extremity injuries in running and associated risk factors: a systematic review. Physical Activity and Health. (2021) 5(1):133–45. 10.5334/PAAH.109

[10] Francis P Whatman C Sheerin K Hume P Johnson MI. The proportion of lower limb running injuries by gender, anatomical location and specific pathology: a systematic review. J Sports Sci Med. (2019) 18(1):21. PMID: 30787648 30787648 PMC6370968

[11] Geraci MC Brown W. Evidence-based treatment of hip and pelvic injuries in runners. Phys Med Rehabil Clin N Am. (2005) 16(3):711–47. 10.1016/j.pmr.2005.02.004 16005401

[12] Taunton JE Ryan MB Clement DB McKenzie DC Lloyd-Smith DR Zumbo BD. A retrospective case-control analysis of 2002 running injuries. Br J Sports Med. (2002) 36(2):95–101. 10.1136/bjsm.36.2.95 11916889 PMC1724490

[13] Ford KR Taylor-Haas JA Genthe K Hugentobler J. Relationship between hip strength and trunk motion in college cross-country runners. Med Sci Sports Exerc. (2013) 45(6):1125–30. 10.1249/MSS.0b013e3182825aca 23274608

[14] Jacobs CA Uhl TL Mattacola CG Shapiro R Rayens WS. Hip abductor function and lower extremity landing kinematics: sex differences. J Athl Train. (2007) 42(1):76–83. PMID: 17597947 17597947 PMC1896084

[15] Leetun DT Ireland ML Willson JD Ballantyne BT Davis IMC. Core stability measures as risk factors for lower extremity injury in athletes. Med Sci Sports Exerc. (2004) 36(6):926–34. 10.1249/01.MSS.0000128145.75199.C3 15179160

[16] Ferber R Davis IMC Williams DS. Gender differences in lower extremity mechanics during running. Clin Biomech. (2003) 18(4):350–7. 10.1016/S0268-0033(03)00025-1 12689785

[17] Schache AG Blanch P Rath D Wrigley T Bennell K. Differences between the sexes in the three-dimensional angular rotations of the lumbo-pelvic-hip complex during treadmill running. J Sports Sci. (2003) 21(2):105–18. 10.1080/0264041031000070859 12630790

[18] Almeida SA Trone DW Leone DM Shaffer RA Patheal SL Long K. Gender differences in musculoskeletal injury rates: a function of symptom reporting? Med Sci Sports Exerc. (1999) 31(12):1807. 10.1097/00005768-199912000-00017 10613432

[19] Kakouris N Yener N Fong DTP. A systematic review of running-related musculoskeletal injuries in runners. In J Sport Health Sci. (2021) 10(5):513–22). 10.1016/j.jshs.2021.04.001 33862272 PMC8500811

[20] Schache AG Bennell KL Blanch PD Wrigley TV. The coordinated movement of the lumbo-pelvic-hip complex during running: a literature review. Gait and Posture. (1999) 10:30–47. 10.1016/S0966-6362(99)00025-9 10469939

[21] Martínez Nova A Blanco Traba M Pérez Soriano P Mosqueira Ourens M López del Amo Lorente A. Dynamic instability of the pelvis and its relation to plantar pressures in runners. Rev Esp Pod. (2020) 31:65–70. 10.20986/revesppod.2020.1558/2020

[22] Seay J Selbie WS Hamill J. In vivo lumbo-sacral forces and moments during constant speed running at different stride lengths. J Sports Sci. (2008) 26(14):1519–29. 10.1080/02640410802298235 18937134

[23] Bramah C Preece SJ Gill N Herrington L. Is there a pathological gait associated with common soft tissue running injuries? Am J Sports Med. (2018) 46(12):3023–31. 10.1177/0363546518793657 30193080

[24] Bramah C Preece SJ Gill N Herrington L. Kinematic characteristics of male runners with a history of recurrent calf muscle strain injury. Int J Sports Phys Ther. (2021) 16(3):732–40. 10.26603/001c.22971 34123526 PMC8169031

[25] Dillon S Burke A Whyte EF Connor SO Gore S Moran KA. Running towards injury? A prospective investigation of factors associated with running injuries. PLoS One. (2023) 18:eo288814. 10.1371/journal.pone.0288814 PMC10434952 37590281

[26] Hennessy L Watson AWS Hennessy L. Flexibility and posture assessment in relation to hamstring injury. Br J Sp Med. (1993) 27:4. 10.1136/BJSM.27.4.243 PMC1332012 8130961

[27] Schuermans J Van Tiggelen D Danneels L Witvrouw E. Susceptibility to hamstring injuries in soccer. Am J Sports Med. (2016) 44(5):1276–85. 10.1177/0363546515626538 26912281

[28] Seay JF Van Emmerik REA Hamill J. Influence of low back pain status on pelvis-trunk coordination during walking and running. Spine. (2011) 36(16):E1070–9. 10.1097/BRS.0b013e3182015f7c 21304421

[29] Novacheck TF. The biomechanics of running. Gait Posture. (1998) 7:77–95. 10200378 10.1016/s0966-6362(97)00038-6

[30] Schache AG Blanch PD Dorn TW Brown NAT Rosemond D Pandy MG. Effect of running speed on lower limb joint kinetics. Med Sci Sports Exerc. (2011) 43(7):1260–71. 10.1249/MSS.0b013e3182084929 21131859

[31] Biewener AA. Biomechanics of mammalian terrestrial locomotion. Science. (1990) 250(4984):1097–103. 10.1126/science.2251499 2251499

[32] Dillman CJ. Kinematic analyses of running. Exerc Sport Sci Rev. (1975) 3(1):193–218. 10.1249/00003677-197500030-00010 1175666

[33] Telhan G Franz JR Dicharry J Wilder RP Riley PO Kerrigan DC. Lower limb joint kinetics during moderately sloped running. J Athl Train. (2010) 45(1):16–21. 10.4085/1062-6050-45.1.16 20064043 PMC2808749

[34] Borba EF de Silva ES da Alves LL de Neto ARDS Inda AR Ibrahim BM. Fatigue-Related changes in running technique and mechanical variables after a maximal incremental test in recreational runners. J Appl Biomech. (2024) 40:1–8. 10.1123/jab.2024-0092 39231490

[35] Maas E De Bie J Vanfleteren R Hoogkamer W Vanwanseele B. Novice runners show greater changes in kinematics with fatigue compared with competitive runners. Sports Biomech. (2018) 17(3):350–60. 10.1080/14763141.2017.1347193 28730917

[36] Tazji MK Valizadeh Ghale-Beig A Sadeghi H Koumantakis GA Chrysagis N Abbasi A. Effects of Running-induced Fatigue on the Trunk-pelvis-hip Coordination Variability During Treadmill Running at Different Speeds. (2023). PMID: 37259658 PMC10233221 37259658

[37] Chumanov ES Wall-Scheffler C Heiderscheit BC. Gender differences in walking and running on level and inclined surfaces. Clin Biomech. (2008) 23(10):1260–8. 10.1016/j.clinbiomech.2008.07.011 18774631

[38] Fox SM Naughton JP Haskell WL. Physical activity and the prevention of coronary heart disease. Ann Clin Res. (1971) 3(6):404–32. 10.1161/JAHA.117.007725 4945367

[39] Liguori G Feito Y Fountaine C Roy BA. ACSM’s guidelines for exercise testing and prescription 9th ed. J Can Chiropr Assoc. (2014) 58(3):328. PMCID: PMC4139760

[40] Jesus RS de Batista RÉS Santos VME Ohara D Alves ES da Ribeiro LFP. Exercise duration affects session ratings of perceived exertion as a function of exercise intensity. Percept Mot Skills. (2021) 128(4):1730–46. 10.1177/00315125211018445/FORMAT/EPUB 34039119

[41] Karvonen MJ Kentala E Mustala O. The effects of training on heart rate—a longitudinal study. Ann Med Exp Biol Fen. (1957) 35(3):307–15. 13470504

[42] Capodaglio EM. Comparison between the CR10 Borg’s scale and the VAS (visual analogue scale) during an arm-cranking exercise. J Occup Rehabil. (2001) 11(2):69–74. 11706532 10.1023/a:1016649717326

[43] Neely G Ljunggren G Sylven C Borg G. Comparison between the visual analogue scale (VAS) and the category ratio scale (CR-10) for the evaluation of leg exertion. Int J Sports Med. (1992) 13(2):133–6. 10.1055/S-2007-1021244/BIB 1555902

[44] Bove AM Lynch AD Depaul SM Terhorst L Irrgang JJ Kelley Fitzgerald G. Test-retest reliability of rating of perceived exertion and agreement with 1-repetition Maximum in adults. J Orthop Sports Phys Ther. (2016) 46(9):768–74. 10.2519/JOSPT.2016.6498 27494056

[45] van Sint Jan S. Color Atlas of Skeletal Landmark Definitions. Edinburgh: Churchill Livingstone Elsevier (2007).

[46] Scharl T Frisch M Fuss FK. Gender differences in the dynamics and kinematics of running and their dependence on footwear. Bioengineering. (2024) 11(12):1261. 10.3390/bioengineering11121261 39768079 PMC11674008

[47] Perpiñá-Martínez S Arguisuelas-Martínez MD Pérez-Domínguez B Nacher-Moltó I Martínez-Gramage J. Differences between sexes and speed levels in pelvic 3D kinematic patterns during running using an inertial measurement unit (IMU). Int J Environ Res Public Health. (2023) 20(4):3631. 10.3390/ijerph20043631 PMC9961938 36834324

[48] Vannatta CN Kernozek TW. Sex differences in gluteal muscle forces during running. Sports Biomech. (2021) 20(3):319–29. 10.1080/14763141.2018.1548641 30526380

[49] Willson JD Petrowitz I Butler RJ Kernozek TW. Male and female gluteal muscle activity and lower extremity kinematics during running. Clin Biomech. (2012) 27(10):1052–7. 10.1016/j.clinbiomech.2012.08.008 22948078

[50] Garciá-Pinillos F Garciá-Ramos A Ramírez-Campillo R Latorre-Román P Roche-Seruendo LE. How do spatiotemporal parameters and lower-body stiffness change with increased running velocity? A comparison between novice and elite level runners. J Hum Kinet. (2019) 70(1):25. 10.2478/HUKIN-2019-0036 PMC6942482 31915473

[51] Mendiguchia J Garrues MA Schilders E Myer GD Dalmau-Pastor M. Anterior pelvic tilt increases hamstring strain and is a key factor to target for injury prevention and rehabilitation. Knee Surg Sports Traumatol Arthrosc. (2024) 32(3):573–82. 10.1002/ksa.12045 38391038

[52] Nurse CA Lewis CL Shefelbine SJ. Frontal plane pelvic kinematics during high velocity running: association with hamstring injury history. Phys Ther Sport. (2023) 64:133–9. 10.1016/j.ptsp.2023.10.002 37890340

[53] Boyer ER Derrick TR. Select injury-related variables are affected by stride length and foot strike style during running. Am J Sports Med. (2015) 43(9):2310–7. 10.1177/0363546515592837 26243741

[54] Willson JD Davis IS. Lower extremity mechanics of females with and without patellofemoral pain across activities with progressively greater task demands. Clin Biomech. (2008) 23(2):203–11. 10.1016/j.clinbiomech.2007.08.025 17942202

[55] Bramah C Preece SJ Gill N Herrington L. A 10% increase in step rate improves running kinematics and clinical outcomes in runners with patellofemoral pain at 4 weeks and 3 months. Am J Sports Med. (2019) 47(14):3406–13. 10.1177/0363546519879693 31657964 PMC6883353

[56] Cruz AC de Fonseca ST Araújo VL Carvalho DS da Barsante LD Pinto VA. Pelvic drop changes due to proximal muscle strengthening depend on foot-ankle varus alignment. Appl Bionics Biomech. (2019) 2019:1–12. 10.1155/2019/2018059 PMC6541954 31223335

[57] Lashien SA Abdelnaeem AO Gomaa EF. Effect of hip abductors training on pelvic drop and knee valgus in runners with medial tibial stress syndrome: a randomized controlled trial. J Orthop Surg Res. (2024) 19(1):700. 10.1186/s13018-024-05139-3 39468623 PMC11520670

[58] Carson NM Aslan DH Ortega JD. The effect of forward postural lean on running economy, kinematics, and muscle activation. PLoS One. (2024) 19(5):e0302249. 10.1371/journal.pone.0302249 38809851 PMC11135760

[59] Engeler N Lichtenstein E Faude O Roth R. How downhill and uphill running interfere posture and muscle activity: a descriptive laboratory study. Int J Sports Phys Ther. (2025) 20(8):1186–97. 10.26603/001c.142485 40756801 PMC12317792

[60] Khassetarash A Vernillo G Martinez A Baggaley M Giandolini M Horvais N. Biomechanics of graded running: part II—joint kinematics and kinetics. Scand J Med Sci Sports. (2020) 30(9):1642–54. 10.1111/sms.13735 32485036

[61] Zifchock RA Davis I Higginson J Royer T. The symmetry angle: a novel, robust method of quantifying asymmetry. Gait Posture. (2008) 27(4):622–7. 10.1016/j.gaitpost.2007.08.006 17913499

[62] Lin Y-C Price K Carmichael DS Maniar N Hickey JT Timmins RG. Validity of inertial measurement units to measure lower-limb kinematics and pelvic orientation at submaximal and maximal effort running speeds. Sensors. (2023) 23(23):9599. 10.3390/s23239599 38067972 PMC10708829

[63] Anderson GS. Iliotibial band friction syndrome. Aust J Sci Med Sport. (1991) 23(3):81–3.

[64] Haghighat F Ebrahimi S Rezaie M Shafiee E Shokouhyan SM Motealleh A. Trunk, pelvis, and knee kinematics during running in females with and without patellofemoral pain. Gait Posture. (2021) 89:80–5. 10.1016/j.gaitpost.2021.06.023 34246176

[65] Lloyd-Smith R Clement DB McKenzie DC Taunton JE. A survey of overuse and traumatic hip and pelvic injuries in athletes. Phys Sportsmed. (1985) 13(10):131–41. 10.1080/00913847.1985.11708907 27409756

[66] Futrell EE Gross KD Reisman D Mullineaux DR Davis IS. Transition to forefoot strike reduces load rates more effectively than altered cadence. J Sport Health Sci. (2020) 9(3):248–57. 10.1016/j.jshs.2019.07.006 32444149 PMC7242218

[67] Ferro-Sánchez A Martín-Castellanos A de la Rubia A García-Aliaga A Hontoria-Galán M Marquina M. An analysis of running impact on different surfaces for injury prevention. Int J Environ Res Public Health. (2023) 20(14):6405. 10.3390/ijerph20146405 PMC10378879 37510637

[68] Malisoux L Delattre N Meyer C Gette P Urhausen A Theisen D. Effect of shoe cushioning on landing impact forces and spatiotemporal parameters during running: results from a randomized trial including 800+recreational runners. Eur J Sport Sci. (2021) 21(7):985–93. 10.1080/17461391.2020.1809713 32781913

[69] Vernillo G Giandolini M Edwards WB Morin JB Samozino P Horvais N Millet GY. Biomechanics and physiology of uphill and downhill running. Sports Med. (2017) 47(4):615–29). 10.1007/s40279-016-0605-y 27501719

[70] Fredericson M Moore T. Muscular balance, core stability, and injury prevention for middle- and long-distance runners. Phys Med Rehabil Clin N Am. (2005) 16(3):669–89. 10.1016/j.pmr.2005.03.001 16005399

[71] Xu D Zhou H Quan W Jiang X Liang M Li S. A new method proposed for realizing human gait pattern recognition: inspirations for the application of sports and clinical gait analysis. Gait Posture. (2024) 107:293–305. 10.1016/j.gaitpost.2023.10.019 37926657

[72] de Almeida MO Saragiotto BT Yamato TP Lopes AD. Is the rearfoot pattern the most frequently foot strike pattern among recreational shod distance runners? Phys Ther Sport. (2015) 16(1):29–33. 10.1016/j.ptsp.2014.02.005 24894762

[73] Patoz A Lussiana T Gindre C Malatesta D. A novel kinematic detection of foot-strike and toe-off events during noninstrumented treadmill running to estimate contact time. J Biomech. (2021) 128:110737. 10.1016/j.jbiomech.2021.110737 34517256

[74] Robinson RO Herzog W Nigg BM. Use of force platform variables to quantify the effects of chiropractic manipulation on gait symmetry. J Manipulative Physiol Ther. (1987) 10(4):172–6. 2958572

