# Correction: Alterations in pelvic kinematics with speed, incline, and fatigue in female runners

**DOI:** 10.3389/fspor.2026.2010170

**Published:** 2026-10-08

**Authors:** Jaka Kovše, Irinej Papuga, Miha Drobnič, Ahsen Buyukaslan, Nina Verdel, Vojko Strojnik, Matej Supej

**Affiliations:** Faculty of Sport, University of Ljubljana, Ljubljana, Slovenia

**Keywords:** asymmetry, biomechanics, mixed effect model, pelvic kinematics, rearfoot strike, running related injury

Author Nina Verdel was omitted as an author in the published article. The correct author list reads:

Jaka Kovše, Irinej Papuga, Miha Drobnič, Ahsen Buyukaslan, Nina Verdel, Vojko Strojnik, Matej Supej

The Author contributions statement has been corrected to read:

JK: Software, Writing – original draft, Resources, Data curation. IP: Writing – review & editing, Methodology, Software, Data curation. MD: Conceptualization, Methodology, Writing – review & editing, Investigation. AB: Methodology, Writing – review & editing. NV: Writing – review & editing, Investigation. VS: Conceptualization, Supervision, Writing – review & editing, Investigation. MS: Conceptualization, Funding acquisition, Writing – review & editing, Project administration, Supervision.

The original version of this article has been updated.

